# Staining ability of herbal tea preparations on a nano-filled composite restorative material – an in-vitro study

**DOI:** 10.12688/f1000research.128029.2

**Published:** 2023-07-04

**Authors:** Raj Kumar Narkedamalli, Vidya Saraswathi Muliya, Kalyana Chakravarthy Pentapati

**Affiliations:** 1 Conservative Dentistry & Endodontics, Manipal College of Dental Sciences, Manipal, Manipal Academy of Higher Education, Manipal, Karnataka, 576104, India; 2 Public Health Dentistry, Manipal College of Dental Sciences, Manipal, Manipal Academy of Higher Education, Manipal, Karnataka, 576104, India

**Keywords:** Herbal Tea, Green Tea, Hibiscus Tea, Moringa Tea, Discoloration, Spectrophotometer

## Abstract

**Background**: Discoloration of tooth-colored restorations due to various factors is one of the principal causes behind the failure of aesthetics. There has been an surge in the consumption of herbal beverages in recent times and the dietary factors play a potential role in the discoloration tooth-coloured restorations. This study was done to juxtapose the staining ability of green tea (GT), moringa tea (MT), and hibiscus tea (HT) on a nano-filled composite restorative material.

**Methods**: The study was conducted in-vitro on composite samples prepared using moulds. 112 discs were prepared from Filtek ^TM^ Z350XT composite using a brass mould lined with mylar strips. Samples were divided into GT, MT, HT, and artificial saliva (AS) groups and immersed in freshly prepared beverages for 15 minutes each day for 45 days. Digital reflectance spectrophotometer was utilized to record color at baseline, 30, and 45 days. Repeated-measures ANOVA with a post-hoc Bonferroni test was used to compare groups within each group. ANOVA with a post-hoc Games Howell test was used to compare mean differences in ΔE among the groups.

**Results**: Maximum discoloration was observed in the GT, followed by HT and MT, with the least being in the AS group at the end of 30 and 45 days (P<0.001 and P<0.001) respectively.

**Conclusions**: The universal nano-filled composite material showed clinically detectable discoloration when exposed to Green Tea, Hibiscus Tea, and Moringa Tea which increased with time. Herbal beverages have the potential to cause discoloration of the composite resin which is often the choice of material for anterior aesthetic restorations.

## Introduction

Failure of aesthetics is one of the common causes for replacement of existing restorations. ^1^ Surface or sub-surface changes lead to microleakage resulting in staining of the superficial layer of composite materials contributing to the aesthetic failure of the restoration. ^2^ The discoloration of composites can be due to intrinsic and extrinsic staining. ^3^^,^^4^ Foods and beverages that are a part of everyday diet lead to discoloration of composites either by absorption or adsorption of colorants. ^5^ Nano-fillers and nanoclusters enhance the long-term stability of composite resin material. The composites with filler particle <0.4 μm tend to retain surface polish for a more extended period. ^6^

Tea is a popular and highly accepted beverage among the Indian population. Various health benefits are claimed by the manufacturers of herbal teas like lowering blood pressure, weight loss, boosting of liver health, immunity, antioxidants, antiaging etc. Due to potential benefits, there is an increasing acceptance among the public for these herbal tea preparations. ^7^ Green tea (GT) is a traditional beverage‚ derived from the *Camellia sinensis,* loaded with antioxidants and nutrients. ^7^ Moringa tea (MT) is derived from extracts of *Moringa oleifera,* which has nutrients, vitamins, minerals, proteins, essential amino acids, chlorophyll, omega-3 oils, and many such phytonutrients. ^8^ Hibiscus tea (HT) is abundant in vitamins A and C, and rich in flavonoids and pro-anthocyanins, which are antioxidants. ^9^

The Z350XT Nanofill Universal Restorative composite resin (3M™ ESPE™ Filtek™) is a nano-filled restorative material with good strength and wear resistance. The manufacturer claims superior polishability and improved fluorescence for excellent aesthetics and a wide variety of shades for natural-looking restorations.

Due to the potential role of dietary factors on the discoloration of composite resin restorations, many studies evaluated the staining potential of various tooth-colored restorations. ^10^ However, there is a dearth of research on the effects of herbal tea preparations on the color stability of nano-filled composite resin. Given this background, the present study aimed to compare the staining potential of GT, HT, and MT on universal nano-filled composite restorative material. The null hypothesis was that there would be no significant difference in the staining ability of these herbal tea preparations.

## Methods

### Sample preparation

After obtaining clearance from the Institutional Ethics Committee, Kasturba Medical College and Kasturba Hospital (805/2018), a total of 112 discs from universal nano-filled composite resin material ( **3M™ ESPE™ Filtek™ Z350XT**) were made using a specially designed brass mould. Each sample had dimensions: 8mm in diameter and 4mm in thickness. The resin material was dispensed into the mould, following which a mylar strip was placed on the resin composite surface. A 1 mm thick glass slab was positioned over the mylar strip to standardize the gap between the curing light and sample. The curing time was adjusted as per the manufacturer’s instructions with an output of 1100 mW/cm ^2^ (Blue Phase, Ivoclar). Additional curing was carried out after the sample was retrieved from the mould. Before the baseline color estimation, all the samples were stored in distilled water at 37°C for 24 hours, following which they were arbitrarily categorized into four study groups (n=28). ^11^

The control group used artificial saliva (AS) (Department of Biochemistry, Kasturba Medical College, Manipal, Karnataka, India). Herbal tea preparations used were green tea (GT), moringa tea (MT), and hibiscus Tea (HT) (Gtee Botanical Extracts Pvt. Ltd., Chennai, Tamil Nadu, India).

### Tea preparation

Every day, fresh solutions were made by dipping two tea bags (2g X 2) into 300 ml of boiling water for 3 minutes, as directed by the manufacturer. Before immersing the samples, the teabags were disposed, and the solution was cooled down to a temperature between 60 to 65° Celsius.

### Immersion regimen

The samples were dipped in the freshly prepared tea solutions for 15 minutes/day for 45 days. Following the immersion procedure, samples were kept in artificial saliva at room temperature for the rest of the day.

### Spectrophotometric analysis

A Digital reflectance spectrophotometer (i1 Pro Digital Reflectance Spectrophotometer, X-Rite Inc. MI, USA) and Profile maker Pro 5.0.10 software was used to assess the color of the samples at baseline, 30 and 45 days. ^12^ Snapper or Loop could be used as alternative software to fulfill a similar function. After drying the specimen using blotting paper, each sample was placed on a white backdrop, with the spectrophotometer’s active point set at the centre of the sample. The change in color for individual sample after 30 days and 45 days of immersion regimen was calculated using the following equation:

$${\text{∆E}}^{*}=\left[\left({\text{∆L}}^{*}\right)\;2+\left({\text{∆a}}^{*}\right)\;2+\left({\text{∆b}}^{*}\right)2\right]1/2$$

### Statistical analysis

All analyses were performed using SPSS version 20 (IBM Corp. Released 2011. IBM SPSS Statistics for Windows, Version 20.0. Armonk, NY: IBM Corp). A P-value of <0.05 was considered statistically significant. Repeated-measures ANOVA with a post-hoc Bonferroni test was used to compare groups within each group. ANOVA with a post-hoc Games Howell test was used to compare mean differences in ΔE among the groups at 30 and 45 days. Data can be accessed at Mendeley datasets. ^13^

## Results

Intra-group comparisons showed that baseline values were the lowest, followed by 30 days, with the highest being at 45 days in GT, MT, HT, and control solutions ( Table 1). The inter-group comparison demonstrated significant differences at the end of 30 days (P<0.001) and 45 days (P<0.001), respectively ( Table 2). The post-hoc test illustrated that the highest discoloration was seen in GT followed by HT, MT with the least being in the control group.

**Table 1.** Intra-group comparison of mean ΔE among the four test solutions.

Test solution	Baseline Mean± SD	30 days Mean± SD	45 days Mean± SD	P-value	Post-hoc test
Green tea	25.35±1.05	42.24±1.91	51.36±1.68	<0.001	45>30>B
Moringa tea	25.09±1.09	28.55±1.73	34.19±0.96	<0.001	45>30>B
Hibiscus tea	25.10±0.6	35.35±2.81	49.04±2.39	<0.001	45>30>B
Articial saliva	25.26±0.92	26.84±0.97	30.37±0.85	<0.001	45>30>B

**Table 2.** Inter-group comparison of mean difference in ΔE.

Mean difference ΔE	Green tea (GT) Mean± SD	Moringa tea (MT) Mean± SD	Hibiscus Tea (HT) Mean± SD	Artificial saliva (AS) Mean± SD	P-value	Post-hoc test
30 days	16.89±2.44	3.52±2	10.25±2.78	1.58±0.28	<0.001	GT>HT>MT>AS
45 days	26.01±2.08	9.1±1.38	23.94±2.5	5.11±0.52	<0.001	GT>HT>MT>AS

## Discussion

Discoloration of dental restorative material in the aesthetic zone has been a matter of concern among clinicians and often requires replacement of the restoration. In order to be considered as a clinical success, the restorative materials used in the aesthetic zones must not only provide an initial shade match but also maintain an aesthetic appearance and over the years in the restored tooth.

Nanocomposites utilize nanofiller incorporation into the resin matrix to amplify the mechanical and aesthetic properties. ^6^ The current in-vitro study assessed the staining ability of herbal teas on commonly used composite restorative material Filtek™ Z350XT (3M ESPE). It is a direct restorative nanocomposite, light-cured, and suitable to be used for both anterior and posterior restorations. Monomers like bisphenol A diglycidyl methacrylate (Bis-GMA), ethoxylated bisphenol A glycol dimethacrylate (Bis-EMA), urethane dimethacrylate (UTDMA) and a small portion of a hydrophilic monomer triethylene glycol dimethacrylate (TEGDMA) are also present in its composition. The color change may be related to the nature of this resin matrix. ^14^ Composite resins that can absorb water are also able to absorb other fluids with pigments, which results in discoloration. It is assumed that water acts as a vehicle for stain penetration into the resin matrix. ^15^ Likewise, the filler content might play an important role in the composite color stability. ^16^

Previous literature on the finishing of resin-based restorations demonstrated that highly smooth surfaces could be obtained when restorations were allowed to polymerize in contact with Mylar strips. ^17^ Hence in the current study, samples were prepared using a brass mould lined by mylar strips. ^18^^–^^20^ Researchers proposed numerous techniques of accelerated aging to study the color changes. Various solutions such as tea, coffee, herbal drinks, cola, chlorhexidine, etc., have been advocated over varying time intervals to assess the discoloration on various aesthetic restorative materials. ^21^^–^^23^ The herbal tea preparations included in the current study have gained popularity and increased acceptance due to the inclination towards a healthy lifestyle. Due to the brief contact of these preparations with the oral cavity, the specimens were immersed for 15 minutes per day. The samples were kept in artificial saliva to simulate the oral environment. It is very crucial to emphasize the impossibility of establishing the exact correlation between in-vitro and in-vivo tests, since the oral environment cannot be reproduced in the laboratory. The color change can be assessed both visually and using specific instruments. ^24^ In the current study, the color changes in the composite resin were assessed using a spectrophotometer which is capable of detecting minute variations. ^25^ The application of CIEL *a*b* system along with the correlated color difference metrics have been devised to meliorate the visual interpretation of colorimetric data. This system has also been proven to accurate for the analysis of ΔE* values ^26^ alongwith additional advantages of repeatability and objectivity. ^27^ The variations in L*, a*, and b are denoted by ΔL*, Δa*, and Δb* respectively, where in, L* corresponds to the degree of discoloration of the test samples. The parameter a* stands for red (+a*) and green (-a*), in the contrary, b* represents yellow (+b*) and blue (-b*). ^28^ The value of ΔE* is more crucial compared to the individual values of L*, a* and b*. ^29^

Many factors such as the size of the particle, the form of the organic matrix, percentage of the particle in the matrix, degree of polymerization, finishing and polishing, ^30^ staining material used, etc., have a potential role on the vulnerability of the dental restorative material to staining. ^31^

The composite restorative material in this study had undergone substantial color change due to the immersion in the tea preparations due to the softening potential of the staining solutions ^32^ The composite resin’s physicochemical characteristics help regulate the harmony of the material in response to extrinsic stains, with water sorption being the most important amongst them. ^33^^–^^35^ The hydrophilicity of the resin matrix of the restorative material is associated with the sensitivity of water sorption and solubility behavior of the resin composite materials. ^36^^,^^37^ Microcracks, voids, or interfacial gaps result from elevated levels of osmotic pressure at the matrix-filler interphases and are prone to stain initiation. ^38^^–^^40^

GT had the maximum potential for discoloration, followed by HT and MT, which may be attributed to the tannin content in GT ^41^ and MT ^8^ and the anthocyanin content in HT. ^11^ The exposure time to the staining solutions determines restorative materials color stability. The results of the present study are in accordance with the previous literature that illustrates the increase in discoloration of composites with an increase in the immersion time. ^42^

Color changes that are imperceptible to the human eye are represented by ΔE values between 0 and 2. In contrast, ΔE values between 2 and 3 represent color changes that are only detectable by the human eye on close inspection. For 50% of qualified observers, values more than or equal to 3.3 are visually apparent at a glance and clinically unacceptable. ^43^ In our study, all the tea preparations showed values of more than 3.3, which was clinically detectable.

Our study highlights the staining potential of these newer herbal teas, affecting dental restorations’ longevity. The patients should be made aware of the aesthetic consequences of these preparations on long-term consumption, and the clinicians should be knowledgeable regarding the staining potential.

## Conclusion

The universal nano-filled composite material showed clinically detectable discoloration when exposed to green tea, hibiscus tea, and moringa tea which increased with time. The staining potential increased with the duration of exposure to the tea preparations. Dentists should be aware of the patient’s dietary preferences and their implications on the longevity of composite restorations.

## Data Availability

Mendeley Data: Staining ability of herbal tea preparations on a nano-filled composite restorative material. https://doi.org/10.17632/bzx3fhd6hw.1. ^13^ Data are available under the terms of the Creative Commons Attribution 4.0 International license (CC-BY 4.0).
