# Supplementary material for: Staining ability of herbal tea preparations on a nano-filled composite restorative material – an in-vitro study
Source: F1000Res. 2023 Jul 4;11:1376. Originally published 2022 Nov 24. [Version 2] doi: 10.12688/f1000research.128029.2 (PMC10457558; doi:10.12688/f1000research.128029.2)
Supplement: Peer review history

# Peer review: Reviewer response for version 2

**DOI:** 10.5256/f1000research.151221.r199707

**Published:** 2023-08-30

**Authors:** Zethy Hanum Mohamed Kassim

**Affiliations:** 1 Universiti Teknologi MARA, Shah Alam, Selangor, Malaysia

This *in vitro* study aimed to investigate the staining potential of popular herbal tea beverages which included Green tea, Hibiscus tea and Moringa tea on a representative nano-filled composite using a static immersion model and analysing the colour difference, ΔE at baseline, 30 days, and 45 days. It was found that all the beverages produced time-dependent colour changes of the nanocomposite samples that were clinically perceptible which may affect the aesthetics and perhaps the maintenance required for this type of composite in tea drinkers.

 The study may be improved by addressing the following:

**Literature review:**

 To include citations of contemporary articles, those written within the last 5 years. Reference related to the colour stability of nanocomposite rather than gloss retention would be more appropriate to the study objective which is to measure the staining potential of various types of herbal tea on nanocomposite. A clinical study that has been recently cited in a CPG published by the ADA has reported that nanocomposite may experience unacceptable colour match compared to hybrid composite makes further investigation regarding the colour stability of nanocomposite and the present study to be useful ^1^^,^^2^.

**Methodology:**

 To ensure replicability of the experiment by others, please explain how the sample size was derived and provide details on the curing time of the composite samples.

**Statistical analysis:**

 To add clarity replace ‘compare groups within each group’ with to perform an intra-group comparison of colour difference at 30 and 45 days with baseline.

**Results:**

 To include mean colour difference/delta E as the parameter of interest in the top row of Table 1 and Table 2.

**Discussion:**

 Paragraph 2

 The present study was not designed to differentiate between extrinsic and intrinsic staining and therefore attribution of the colour changes to the nature of the resin or filler loading which generally contributes to intrinsic staining may not be relevant ^3^. It may be of interest to the readers for authors to elaborate on the mechanism of extrinsic staining, especially by tea beverages, of which the present study is designed to measure.

 Paragraph 3

 Curing of the composite samples under a glass plate in this experiment would have led to a resin-rich surface layer which can adsorb more stain and contribute to the high delta E value in the present study ^3^^,^^4^.

Is the work clearly and accurately presented and does it cite the current literature?

Partly

If applicable, is the statistical analysis and its interpretation appropriate?

Yes

Are all the source data underlying the results available to ensure full reproducibility?

Yes

Is the study design appropriate and is the work technically sound?

Yes

Are the conclusions drawn adequately supported by the results?

Yes

Are sufficient details of methods and analysis provided to allow replication by others?

Partly

Reviewer Expertise:

Dental Materials, and Prosthodontics

I confirm that I have read this submission and believe that I have an appropriate level of expertise to confirm that it is of an acceptable scientific standard, however I have significant reservations, as outlined above.

## References

[1] : Evidence-based clinical practice guideline on restorative treatments for caries lesions. *The Journal of the American Dental Association* .2023;154(7) : 10.1016/j.adaj.2023.04.011 551-566.e51 10.1016/j.adaj.2023.04.011 37380250

[2] : A 12-month clinical evaluation of composite resins in class III restorations. *J Adhes Dent* .2007;9(1) :57-64 17432402

[3] : Thermocycling Stain Challenge and Colour Stability of two Current Zirconium Silicate-filled Indirect Composite Restorative Materials. *The Open Dentistry Journal* .2023;17(1) : 10.2174/18742106-v17-e230510-2022-169 10.2174/18742106-v17-e230510-2022-169

[4] : Relation of finish to discoloration of composite resins. *The Journal of Prosthetic Dentistry* .1984;52(6) : 10.1016/S0022-3913(84)80010-4 811-814 10.1016/S0022-3913(84)80010-4 6595392

# Peer review: Reviewer response for version 2

**DOI:** 10.5256/f1000research.151221.r183944

**Published:** 2023-08-25

**Authors:** Abhishek Parolia

**Affiliations:** 1 Department of Endodontics, College of Dentistry and Dental Clinics, The University of Iowa, Iowa City, Iowa, USA

I have gone through the revision and I recommend the Approval of this manuscript.

Is the work clearly and accurately presented and does it cite the current literature?

Partly

If applicable, is the statistical analysis and its interpretation appropriate?

I cannot comment. A qualified statistician is required.

Are all the source data underlying the results available to ensure full reproducibility?

Partly

Is the study design appropriate and is the work technically sound?

Yes

Are the conclusions drawn adequately supported by the results?

Yes

Are sufficient details of methods and analysis provided to allow replication by others?

Partly

Reviewer Expertise:

NA

I confirm that I have read this submission and believe that I have an appropriate level of expertise to confirm that it is of an acceptable scientific standard.

# Peer review: Reviewer response for version 2

**DOI:** 10.5256/f1000research.151221.r183943

**Published:** 2023-07-12

**Authors:** Deepshikha Chowdhury

**Affiliations:** 1 Private Practitioner, Kolkata, West Bengal, India

The responses that have been submitted are satisfactory to me. The article maybe forwarded for indexing.

Is the work clearly and accurately presented and does it cite the current literature?

Yes

If applicable, is the statistical analysis and its interpretation appropriate?

Yes

Are all the source data underlying the results available to ensure full reproducibility?

Yes

Is the study design appropriate and is the work technically sound?

Yes

Are the conclusions drawn adequately supported by the results?

Yes

Are sufficient details of methods and analysis provided to allow replication by others?

Yes

Reviewer Expertise:

Dental materials, endodontics

I confirm that I have read this submission and believe that I have an appropriate level of expertise to confirm that it is of an acceptable scientific standard.

# Peer review: Reviewer response for version 1

**DOI:** 10.5256/f1000research.140583.r167509

**Published:** 2023-05-22

**Authors:** Abhishek Parolia

**Affiliations:** 1 Department of Endodontics, College of Dentistry and Dental Clinics, The University of Iowa, Iowa City, Iowa, USA

I have given my suggestions in the attached PDF. This manuscript needs major revision before acceptance for indexing.

Is the work clearly and accurately presented and does it cite the current literature?

Partly

If applicable, is the statistical analysis and its interpretation appropriate?

I cannot comment. A qualified statistician is required.

Are all the source data underlying the results available to ensure full reproducibility?

Partly

Is the study design appropriate and is the work technically sound?

Yes

Are the conclusions drawn adequately supported by the results?

Yes

Are sufficient details of methods and analysis provided to allow replication by others?

Partly

Reviewer Expertise:

NA

I confirm that I have read this submission and believe that I have an appropriate level of expertise to confirm that it is of an acceptable scientific standard, however I have significant reservations, as outlined above.

# Response

**Published:** 2023-06-06

**Authors:** Vidya Saraswathi Muliya

**Affiliations:** Manipal College of Dental Sciences, Manipal, Manipal Academy of Higher Education, India

1: Is the institutional ethical clearance taken? That needs to be stated.

 Response: It has been added in Methods Paragraph 1

 2: How was the sample size calculated?

 Response: Sample size calculation was done using Gpower software (version 3.1.9.4). The effect size of 0.4 (large effect size) was considered for a priori sample size calculation with maximum power (95%) and alpha of 5% for four groups. The total sample size was estimated to be 112 (n=28 per group)

 3: How many investigators prepared all samples?

 Response: One investigator 

 4: material for the mould?

 Response: Brass

 5: this reference is for what?

 Response: This reference is to justify the storage of samples in distilled water prior to initiation of the experiment

 6: More information of the extract is needed in the methodology

 Response: Details are mentioned in the discussion

 7: How did this simulate the real scenarios? Exposure time of a restored tooth to the tea in mouth may not be continuous for 15 minutes.

 Response: The average consumption of tea is around 3-4 cups per day. This can however vary from person to person in real life scenario. So, considering the average tea consumption and a time of 5-7 minutes per cup of tea, the immersion time period is considered as 15 minutes per day in the current study (Patil *et al.*, Clinical Cosmetic & Investigational Dentistry, 2020). The conditions however cannot be completely related to real life (in-vivo) scenario since the exact amount and time of tea consumption varies from person to person, in addition to factors like salivary clearance, dietary habits etc. which may have influence of discoloration

 8. There is no different in the data when presented in the table or figure so choose either of these two

 Response: Figure 1 has been deleted

 9: Company name and country for Spectrophotometer?

 Response: Digital reflectance spectrophotometer - ( i1 Pro Digital Reflectance Spectrophotometer, X-Rite Inc. MI, USA) and Profile maker Pro 5.0.10 software

 10: Discussion needs to be improved and justify the results in more detail

 Response: Discussion has been revised

 11: It is really important to know the exact composition of this tea and which component affects the outcome

 Response: This has been mentioned in the discussion as ‘GT had the maximum potential for discoloration, followed by HT and MT, which may be attributed to the tannin content

 in GT and MT and the anthocyanin content in HT.’

 12: Role of every authors needs to be written

 Response: Mentioned in page 2 of uploaded manuscript

 13: Any funding for this project?

 Response: No funding received. Mentioned in page 2 of uploaded manuscript

# Peer review: Reviewer response for version 1

**DOI:** 10.5256/f1000research.140583.r156562

**Published:** 2022-12-13

**Authors:** Deepshikha Chowdhury

**Affiliations:** 1 Private Practitioner, Kolkata, West Bengal, India

I think this paper is excellent and well written. I really like the conceptualisation of analysing the staining ability of herbal tea preparation that have become a popular beverage in India as well as internationally. It is an important addition to literature. It has been observed that the spectrophotometric analysis has been described in details. Statistical analysis has been done meticulously which is really appreciable. The use of recent citations has increased the credibility of the article. However, I have a few queries that require clearance. They have been mentioned as follows:

1. How was the sample size calculated?
2. Why was the 15 mins time of immersion selected as it doesn't simulate the natural intake of beverage? This can lead to a difference in case of an *in vivo* scenario.
3. Why 4 mm thickness of the samples have been selected? The general increment while curing with LED light is 2 mm unless the composite is a bulk fill.

Is the work clearly and accurately presented and does it cite the current literature?

Yes

If applicable, is the statistical analysis and its interpretation appropriate?

Yes

Are all the source data underlying the results available to ensure full reproducibility?

Yes

Is the study design appropriate and is the work technically sound?

Yes

Are the conclusions drawn adequately supported by the results?

Yes

Are sufficient details of methods and analysis provided to allow replication by others?

Yes

Reviewer Expertise:

Dental materials, endodontics

I confirm that I have read this submission and believe that I have an appropriate level of expertise to confirm that it is of an acceptable scientific standard, however I have significant reservations, as outlined above.

# Response

**Published:** 2023-01-22

**Authors:** Vidya Saraswathi Muliya

**Affiliations:** Manipal College of Dental Sciences, Manipal, Manipal Academy of Higher Education, India

1. How was the sample size calculated?

**Response:** Sample size calculation was done using Gpower software (version 3.1.9.4). The effect size of 0.4 (large effect size) was considered for a priori sample size calculation with maximum power (95%) and alpha of 5% for four groups. The total sample size was estimated to be 112 (n=28 per group)

 2. Why was the 15 mins time of immersion selected as it doesn't simulate the natural intake of beverage? This can lead to a difference in case of an *in vivo* scenario.

**Response:** The average consumption of tea is around 3-4 cups per day. This can however vary from person to person in real life scenario. So, considering the average tea consumption and a time of 5-7 minutes per cup of tea, the immersion time period is considered as 15 minutes per day in the current study (Patil *et al.*, Clinical Cosmetic & Investigational Dentistry, 2020). The conditions however cannot be completely related to real life (in-vivo) scenario since the exact amount and time of tea consumption varies from person to person, in addition to factors like salivary clearance, dietary habits etc. which may have influence of discoloration.

 3. Why 4 mm thickness of the samples have been selected? The general increment while curing with LED light is 2 mm unless the composite is a bulk fill.

**Response:** Samples were prepared using 4mm thickness molds. Handling was easier with 4mm thickness. Since a universal composite material was used in the current study, which had a curing depth of 2mm only, samples were cured equally from both the sides as per the manufacturer’s instructions.
